# Supplementary material for: Adaptive and multifunctional hydrogel hybrid probes for long-term sensing and modulation of neural activity
Source: Nat Commun. 2021 Jun 8;12:3435. doi: 10.1038/s41467-021-23802-9 (PMC8187649; doi:10.1038/s41467-021-23802-9)
Supplement: Supplementary file 1 — Supplementary information [file 41467_2021_23802_MOESM1_ESM.pdf]

# Adaptive and Multifunctional Hydrogel Hybrid Probes for Long-Term Sensing and Modulation of Neural Activity

Park et al.

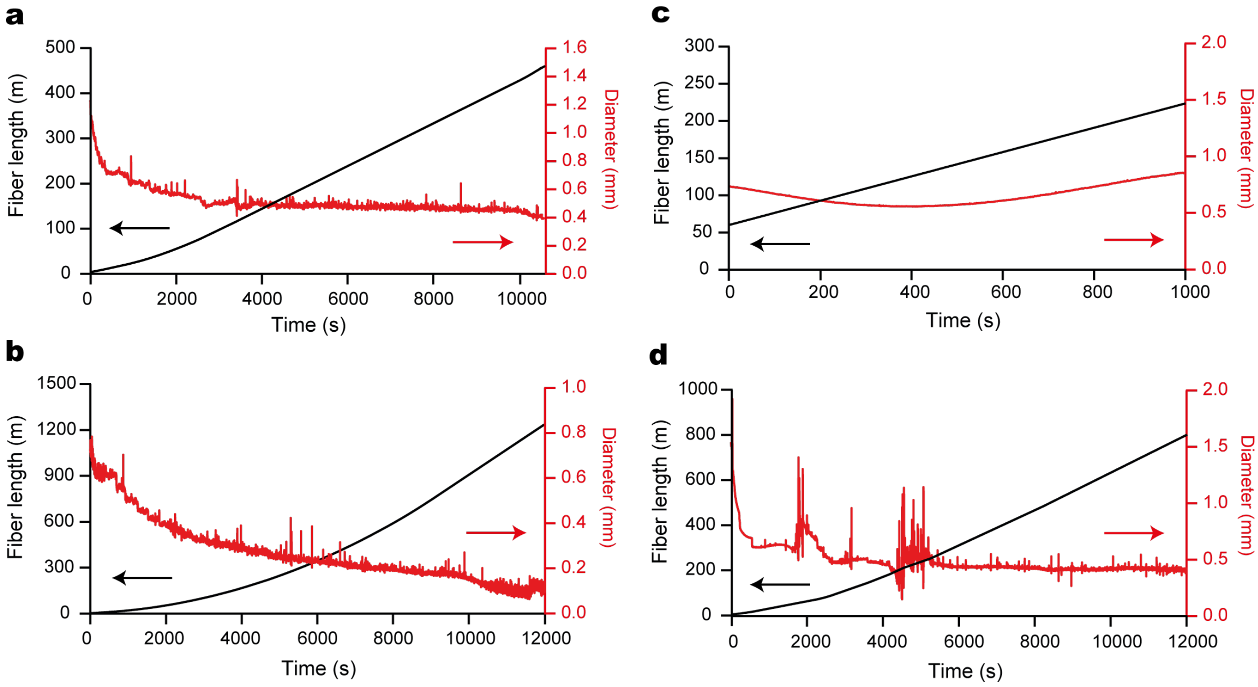


**Supplementary Fig. 1 | Size-tunability and high throughput of the thermal drawing process. a-d**, The length and the diameter of optical waveguide (a), microfluidic channel (b), and microelectrode array first (c) and second (d) drawing steps, measured during the drawing process over time. Feed speed is 0.6 mm $min^{-1}$. Diameters of the optical waveguide and microelectrode array fibers include the thickness of sacrificial layers.

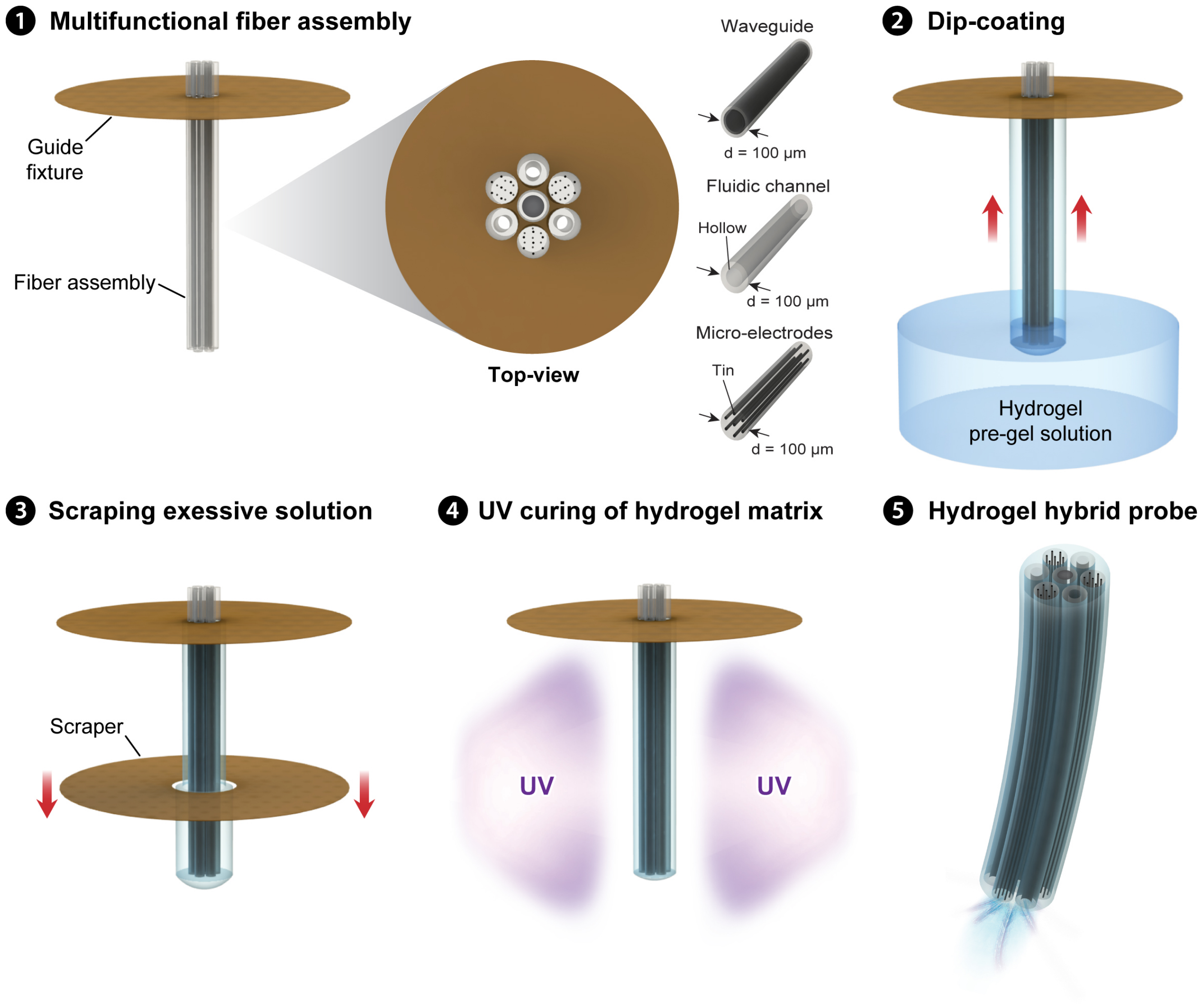


**Supplementary Fig. 2 | Illustration for fabrication of hydrogel hybrid probes. (1)** Individual functional fibers fabricated via thermal drawing are assembled within a polyimide guide fixture, and then connected to the external terminals (optical ferrules, electrical pin connectors, and fluidic tubing). The probes used in this study consist of one waveguide at the center (PC core and COC cladding, 105.9±8.0 µm diameter), three microelectrode arrays (7 Tin microwire (4.75±2.22 µm diameter) encapsulated PEI insulating cladding (80.0±1.8 µm diameter), and three microfluidic channels (PEI microtube, 54.0±2.1 µm inner and 115.4±3.0 µm outer diameters) in an alternating concentric arrangement; (2) The surfaces of the fibers within the assembly are functionalized with primary amines and subsequently alginate, and then dip-coated with the hydrogel pre-gel solution; (3) Excessive pre-gel solution is scraped off with a polyimide scraper to ensure the uniform thickness (25 µm) of hydrogel matrix around the polymer fiber assembly; (4) The hydrogel pre-gel solution is cured by ultraviolet (UV) irradiation to form a soft tough hydrogel matrix; (5) The resultant hydrogel hybrid probe is thoroughly washed with PBS before implantation to remove unreacted reagents.

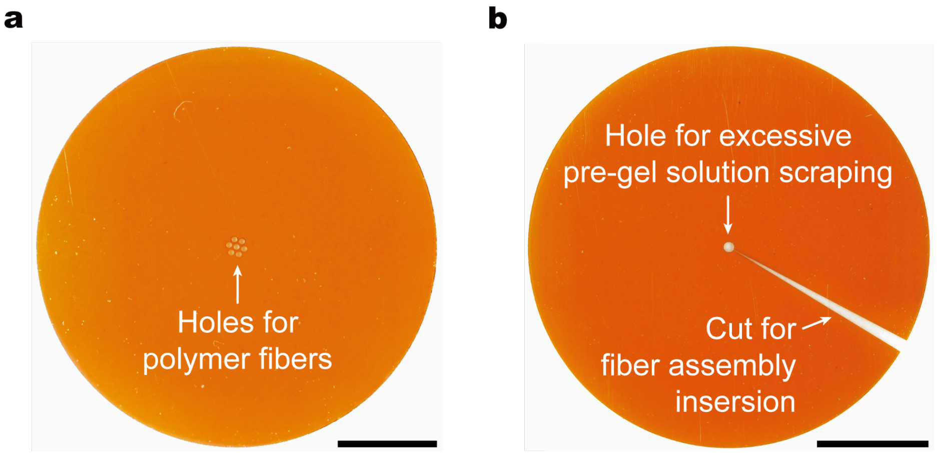


**Supplementary Fig. 3 | A guide fixture and a scraper for the fabrication of hydrogel hybrid probes.** **a**, Guide fixture for functional fiber assembly. Scale bar: 2 mm. **b**, Scraper for the excess hydrogel removal following the hydrogel dip-coating process to ensure the uniform thickness of hydrogel matrix around the polymer fiber assembly (hydrogel thickness: 25 μm). Scale bar: 5 mm.

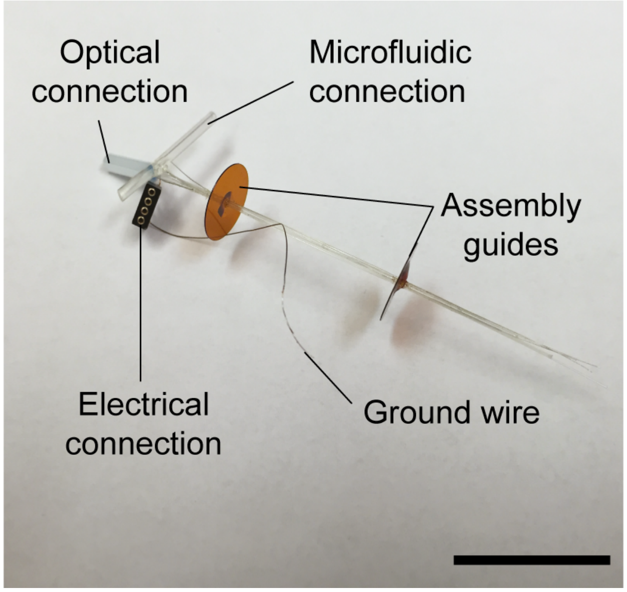


**Supplementary Fig. 4 |** A Photograph of the multifunctional fiber assembly including the guide fixture connectorized to external electrical, optical, and fluidic interfaces prior to the hydrogel coating. Scale bar: 2 cm.

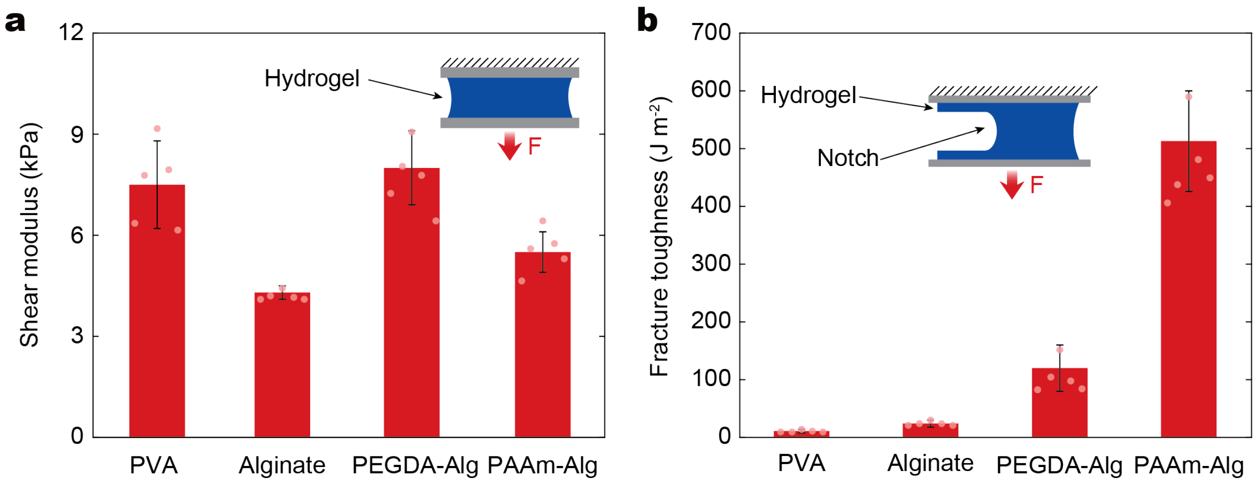


**Supplementary Fig. 5 | Mechanical properties of various candidate hydrogels for probe matrix. a**, Shear moduli of various candidate hydrogels for probe matrix in fully swollen state (in DPBS). **b**, Fracture toughness of various candidate hydrogels for probe matrix in fully swollen state (in DPBS). Values in **a**,**b** represent the mean and the standard deviation ($n$ = 5).

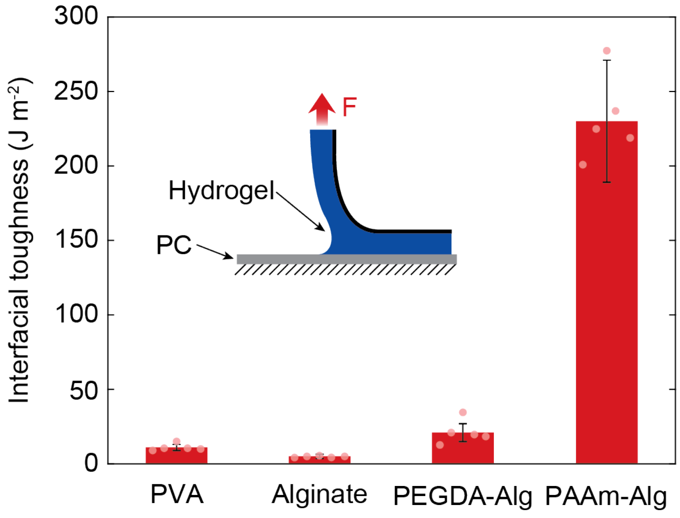


**Supplementary Fig. 6 | Adhesion performance of various candidate hydrogels for probe matrix on surface functionalized PC.** PC substrates are surface functionalized with primary amine for PVA hydrogel and alginate for alginate, PEGDA-Alg, and PAAm-Alg hydrogels. Values represent the mean and the standard deviation ($n$ = 5).

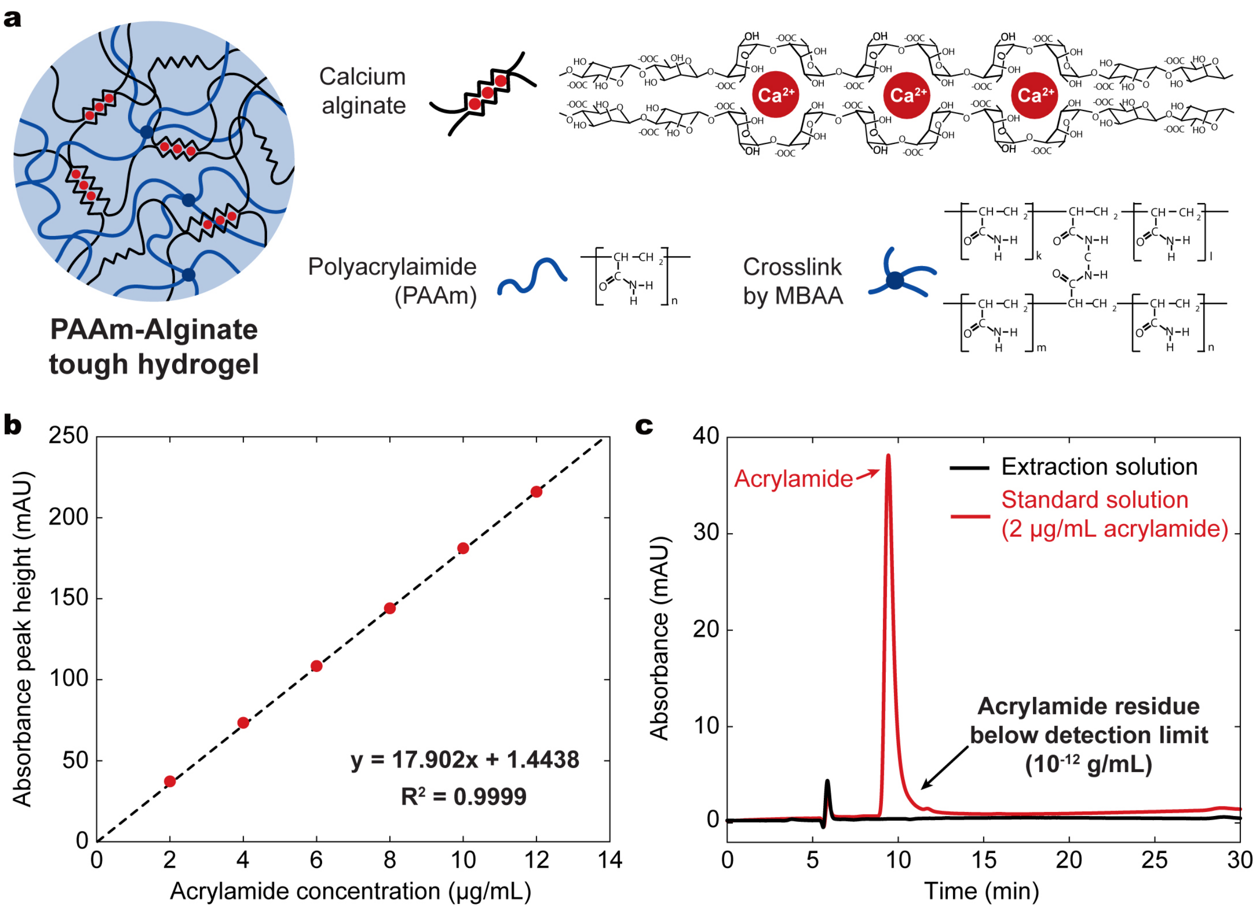


**Supplementary Fig. 7 | Chemical composition and purity of PAAm-Alg tough hydrogel matrix. a**, Chemical composition of poly(acrylamide)-alginate (PAAm-Alg) tough hydrogel matrix for hydrogel hybrid probes. **b**, Standard calibration curve of acrylamide for HPLC. **c**, HPLC characterization results for the PAAm-Alg hydrogel extraction solution and the standard solution with 2 μg $mL^{-1}$ acrylamide. The PAAm-Alg hydrogel extraction solution does not show detectable residual acrylamide (below the detection limit of $10^{-12}$ g $mL^{-1}$).

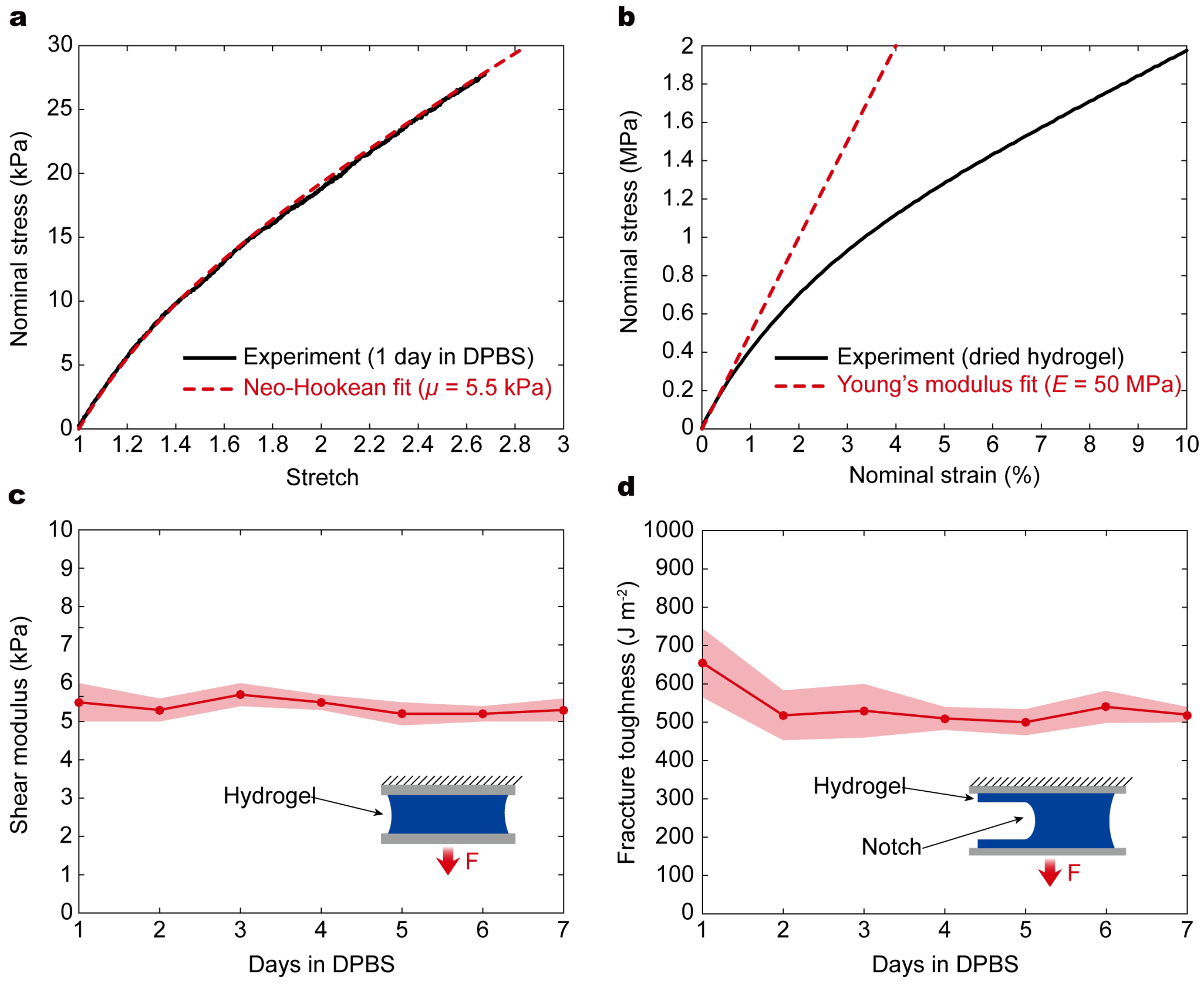


**Supplementary Fig. 8 | Mechanical properties of PAAm-Alg hydrogel. a**, Stretch vs. nominal stress curve of PAAm-Alg hydrogel soaked in DPBS for 1 day. Dotted line is fitting for neo-Hookean model with shear modulus, $\mu$ of 5.5 kPa. **b**, Stretch vs. nominal stress curve of PAAm-Alg hydrogel in a fully dehydrated state. Dotted line indicates Young's modulus, $E$ of 50 MPa. **c**, Changes in shear moduli of PAAm-Alg hydrogel over varying soaking time in DPBS. **d**, Changes in fracture toughness of PAAm-Alg hydrogel over varying soaking time in DPBS[1]. Lines and shaded areas in **c**,**d** represent the mean and the standard deviation, respectively ($n$ = 5).

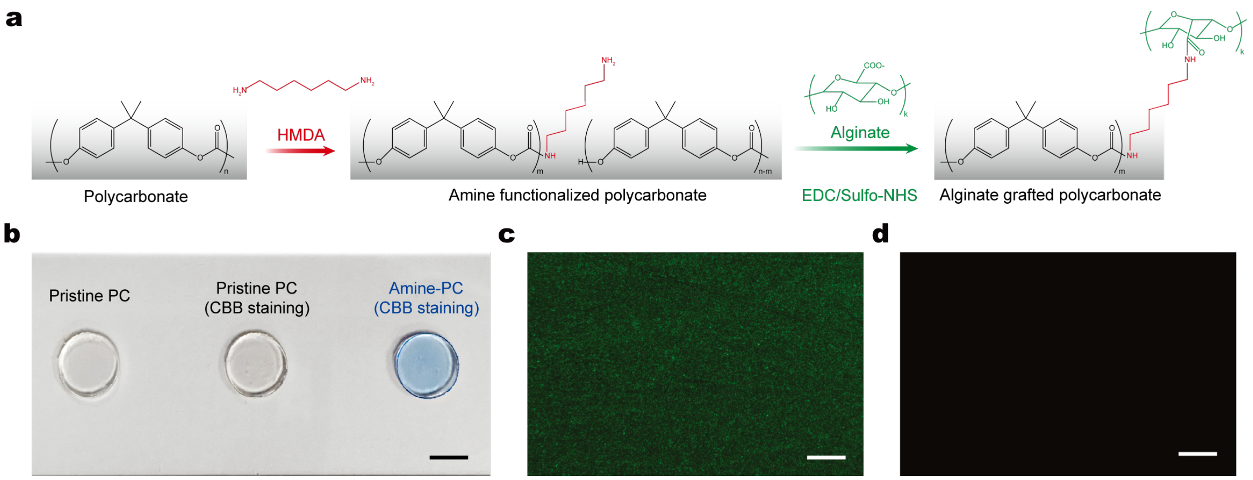


**Supplementary Fig. 9 | Surface functionalization for polymer fiber surface.** **a**, A schematic of polymer fiber surface functionalization for robust hydrogel bonding[2] (shown for PC and similar chemistry applies to PEI). **b**, A photograph of the PC substrates with and without primary amine functionalization by HMDA. Coomassie Brilliant Blue (CBB) staining is used to indicate the presence of primary amine groups[3]. Scale bar: 5 mm. **c**,**d**, Fluorescent microscope images of the PC substrates grafted (c) and not grafted (d) with alginate. Covalently coupled amino-fluorescein (green fluorescence) is used to indicate the presence of surface grafted alginate[4]. The experiment was repeated three times independently for each group with the similar results. Scale bars: 20 µm.

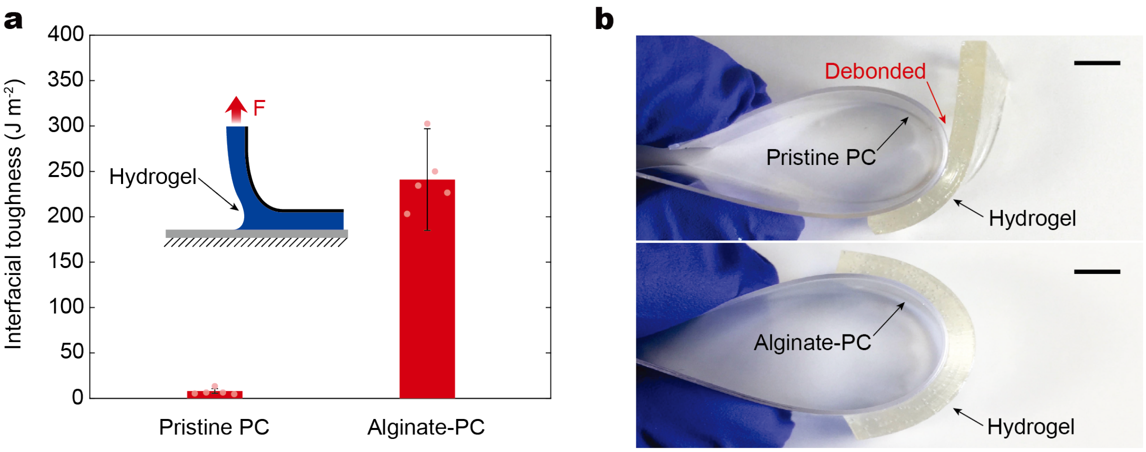


**Supplementary Fig. 10 | Robust bonding of PAAm-Alg hydrogel onto polymer surfaces. a**, Experimentally measured interfacial toughness of PAAm-Alg hydrogel on the pristine and alginate-grafted PC[5]. **b**, Photographs of PAAm-Alg hydrogel prepared on the pristine and alginate-grafted PC under bending deformation. Scale bars: 10 mm. Values in **a** represent the mean and the standard deviation ($n$ = 5).

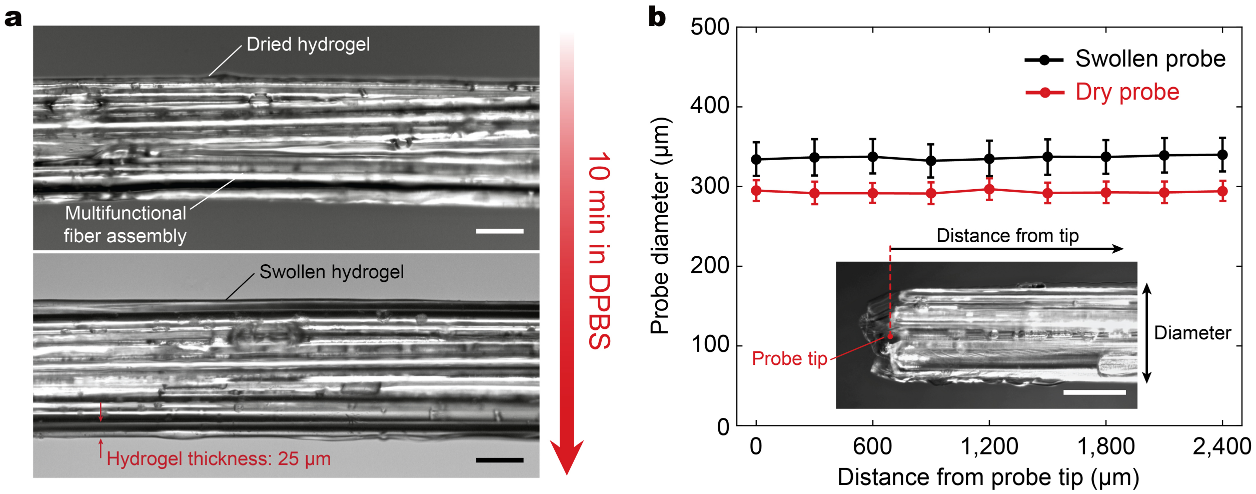


**Supplementary Fig. 11 | Swelling and dimension of hydrogel hybrid probes.** **a**, Swelling of a dried hydrogel hybrid probe within 10 min in DBPS. Scale bars: 200 μm. **b**, Diameter of the dry and swollen hydrogel hybrid probes as a function of distance from the probe tip. Scale bar: 200 μm. Values in **b** represent the mean and the standard deviation ($n = 4$).

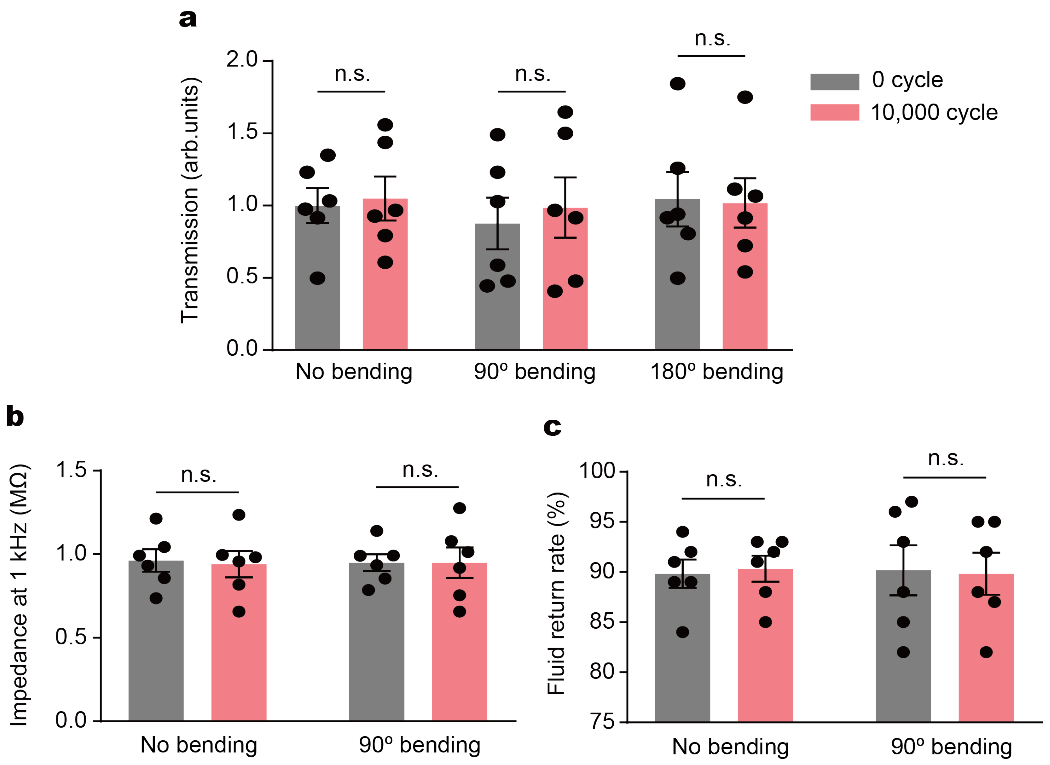


**Supplementary Fig. 12 | Characterizations of the hydrogel hybrid probes following 10,000 cycles of 90° bending deformation at a radius of curvature $R_c$ = 2 mm. a**, Relative optical transmission rate of the hydrogel hybrid probes before/after the repeated cyclic deformation. Measurements are performed at 0°, 90°, and 180° bending deformation ($R_c = 2$ mm). **b**, Impedance of the electrodes at 1 kHz in the hydrogel hybrid probes before/after the repeated cyclic test. Measurements are performed at 0° and 90° bending deformation ($R_c = 2$ mm). **c**, Fluid return rate of the hydrogel hybrid probes before/after the repeated cyclic test. Measurements are performed at 0° and 90° bending deformation ($R_c = 2$ mm). Two-way ANOVA and Sidak's multiple comparison test are performed. Optical test: no bending: $p = 0.9092$, 90° bending: $p = 0.4692$, 180° bending: $p = 0.9845$. Electrical test: no bending: $p = 0.9778$, 90° bending: $p > 0.9999$. Fluidic test: no bending: $p = 0.9659$, 90° bending: $p = 0.9847$. Values in **a-c** represent the mean and the standard deviation ($n = 6$).

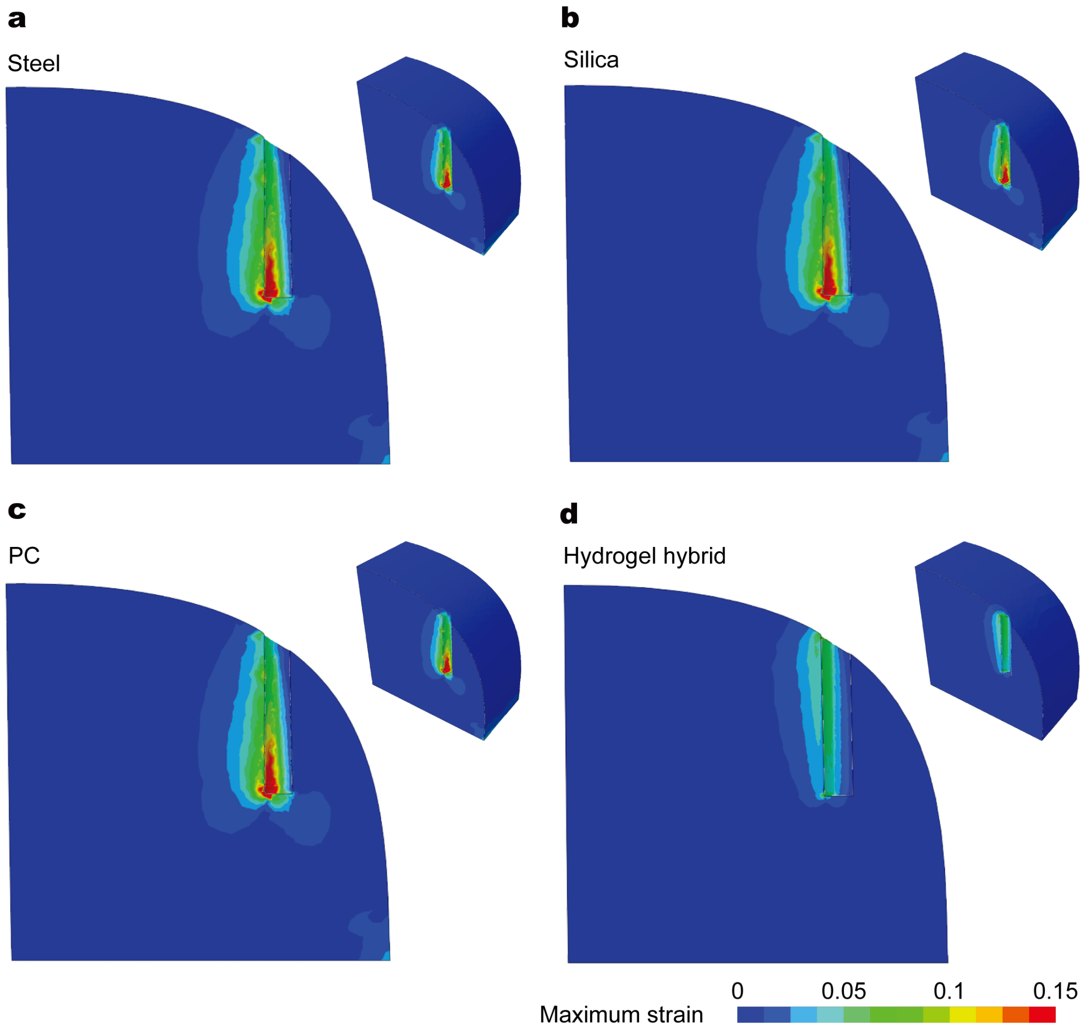


**Supplementary Fig. 13 | Maximum strain profiles within the brain tissue during 100 µm lateral micromotion.** **a**, Stainless steel fiber. **b,** Silica fiber. **c,** PC fiber. **d,** Hydrogel hybrid probe. Inset images represent 3D isometric view of the same FEA results.

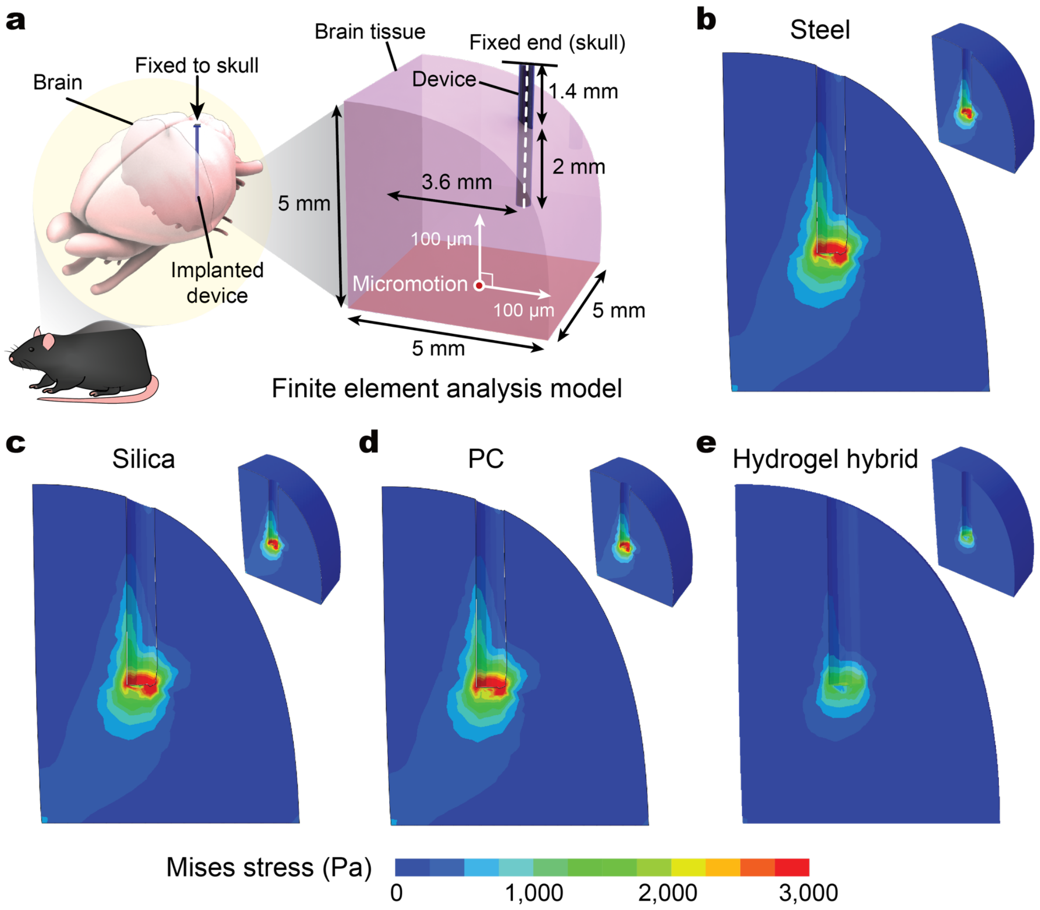


**Supplementary Fig. 14 | Mises stress profiles during 100 µm lateral and 100 µm vertical mixed micromotion. a**, A schematic illustration for the FEA model of the implanted device fixed to the skull during the lateral and vertical mixed brain micromotion. **b-e**, Mises stress profiles within the brain tissue for stainless steel (b), silica (c), PC (d) and the swollen hydrogel hybrid probe (e) at 100 µm lateral and 100 µm vertical micromotion of the brain tissue. Inset images represent 3D isometric view of the same FEA results.

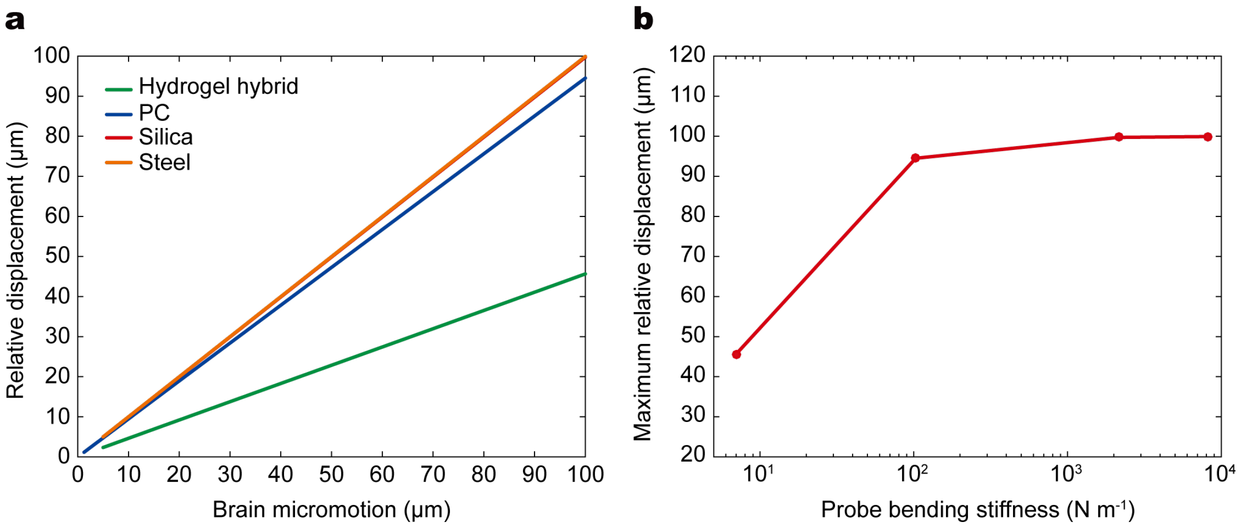


**Supplementary Fig. 15 | Relative displacement between the probes and the brain tissue during micromotion. a**, The relative displacement between various probes and the brain tissue during 0 – 100 μm of lateral micromotion of the brain tissue. **b**, The maximum relative displacement as a function of bending stiffness of the probes.

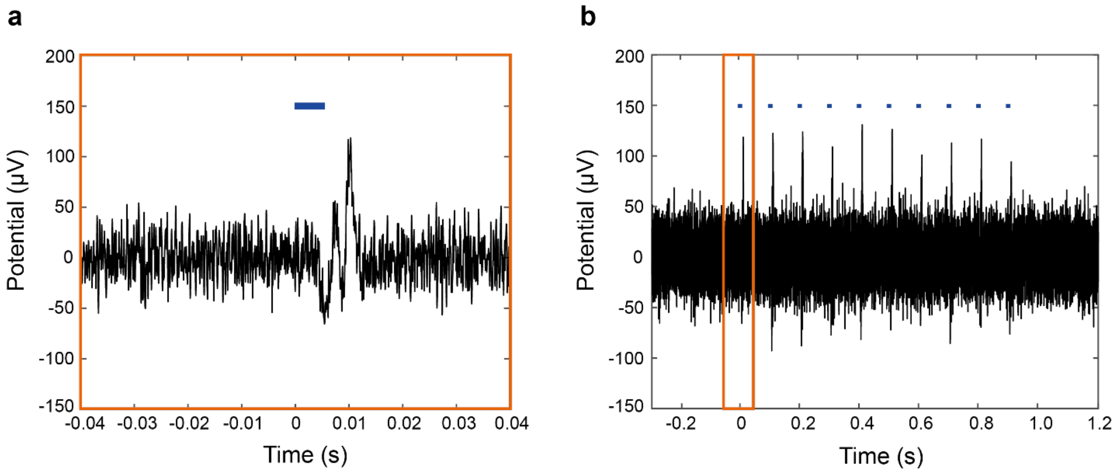


**Supplementary Fig. 16 | Optically-evoked potentials in a large time scale. a**,**b**, Representative electrophysiological recordings in the vHPC during optical stimulation (10 Hz, 10 mW mm$^{-2}$, 5 ms pulse width) from the hydrogel hybrid probes 4 weeks following the implantation and transfection with AAV5-CaMKIIα::ChR2-eYFP with time window of 0.1 second (a) and 1.5 second (b). The orange box in **b** represents the data shown in **a**.

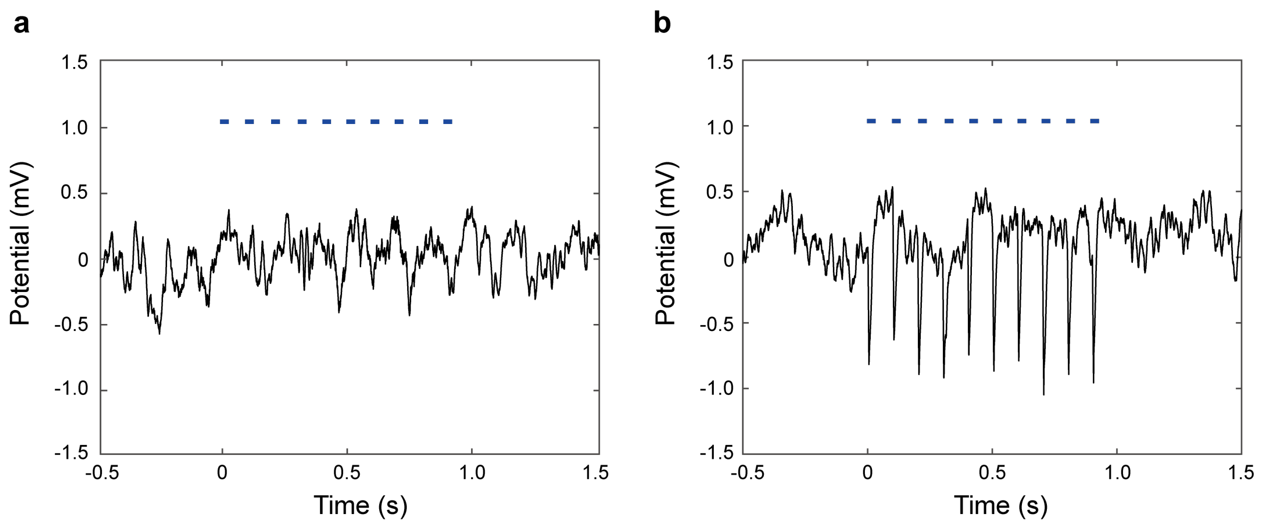


**Supplementary Fig. 17 | Local field potentials (LFPs) recorded by hydrogel-hybrid probes.** **a**,**b**, LFP recording in the vHPC during the optical stimulation (10 Hz, 10 mW $mm^{-2}$, 5 ms pulse width) 4 weeks following the implantation and transfection with AAV5-CaMKIIα::eYFP (a) and AAV5-CaMKIIα::ChR2-eYFP (b).

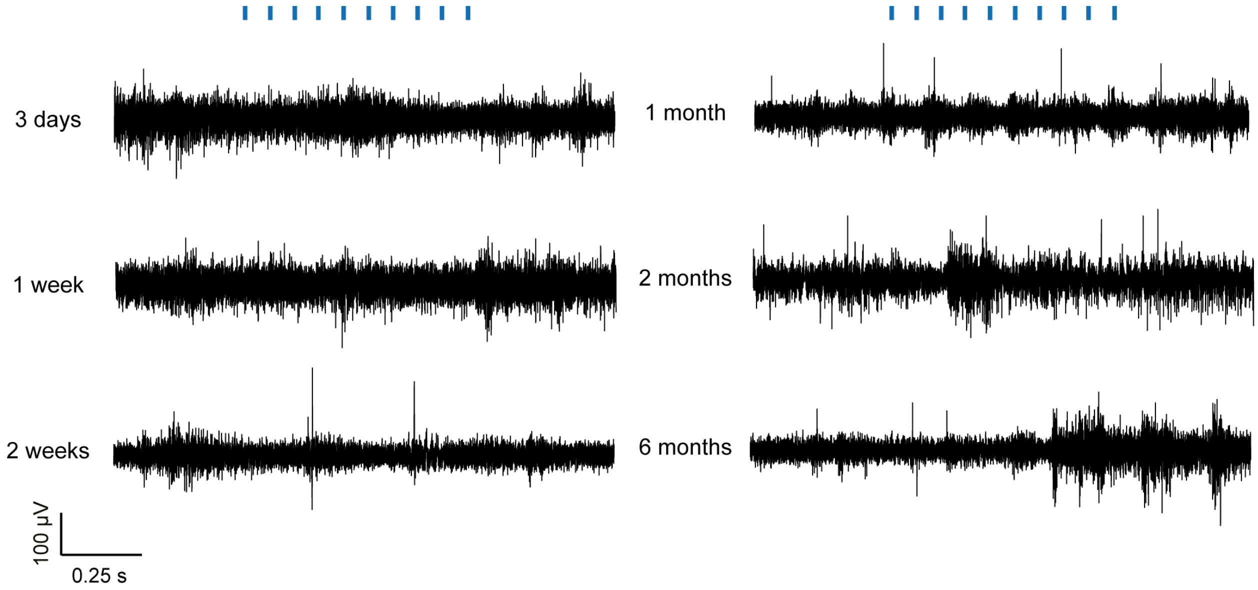


**Supplementary Fig. 18 | Long-term opto-electrophysiological recording in mice injected with the control virus.** Electrophysiological recording in the vHPC during the optical stimulation of the BLA-to-vHPC projections using the hydrogel hybrid probes from 3 days to 6 months following the transfection with AAV5-CaMKIIα::eYFP in the BLA. Optical parameters: 10 Hz, 473 nm, 10 mW $mm^{-2}$, 5 ms pulse width.

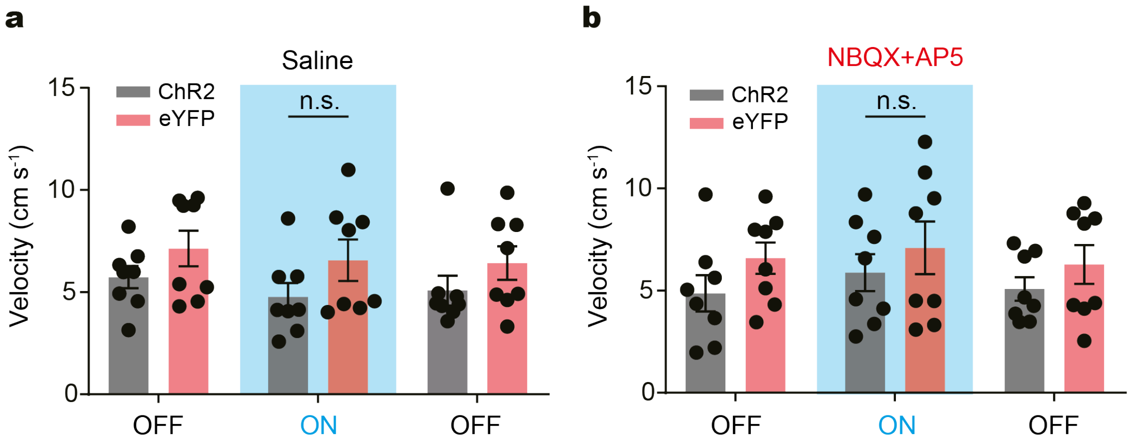


**Supplementary Fig. 19 | Investigation of locomotor effects of optogenetic stimulation of the BLA-to-vHPC projection circuit. a**,**b**, Mouse velocity in the open field during the optical stimulation (20 Hz, 10 mW mm$^{-2}$, 5 ms pulse width, OFF/ON/OFF cycle, 3 min epoch). Velocities are measured for mice expressing ChR2-eYFP and eYFP control (a) in the absence or (b) following the injection of the combination of glutamate receptor antagonist cocktail (AMPA and NMDA receptor antagonist, NBQX+AP5). No statistical difference is found for both groups during the optical stimulation (two-way ANOVA with Bonferroni multiple comparison test, saline: $p$ = 0.1804, drug: $p \geq 0.9999$). Values in **a**,**b**, represent the mean and the standard deviation ($n$ = 8).

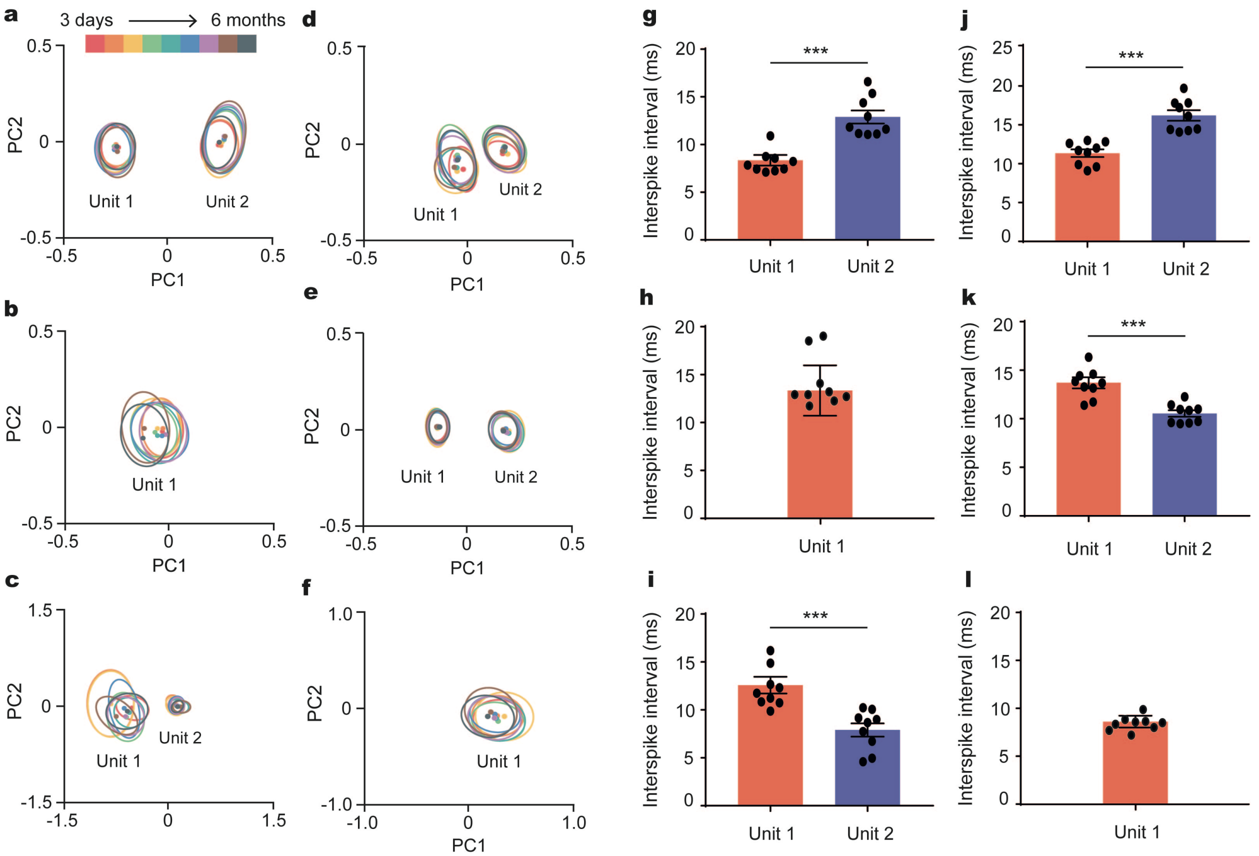


**Supplementary Fig. 20 | Long-term investigation of single-unit potentials. a-f**, Principle component analysis of the recorded single-unit potentials from electrode D (a), electrode H (b), electrode L (c), electrode M (d), electrode O (e), and electrode Q (f) 3 days, 1 week, 2 weeks, 1 month, 2 months, 3 months, 4 months, 5 months, 6 months following the hybrid probe implantation. Markers are the center of principle component clusters and ovals are 2σ contour of the cluster distribution. L-ratio: 0.00021 (a), 0.00063 (c), 0.00407 (d), 0.00025 (e). **g-l**, Average inter-spike intervals of each unit from electrode D (g), electrode H (h), electrode L (i), electrode M (j), electrode O (k), and electrode Q (l). Values in **g-l** represent the mean and the standard deviation ($n$ = 9, independent time point). Significant difference is confirmed by one-way Student's t test. P values: 0.00004 (g), 0.0003 (i), 0.00006 (j), 0.0001 (k).

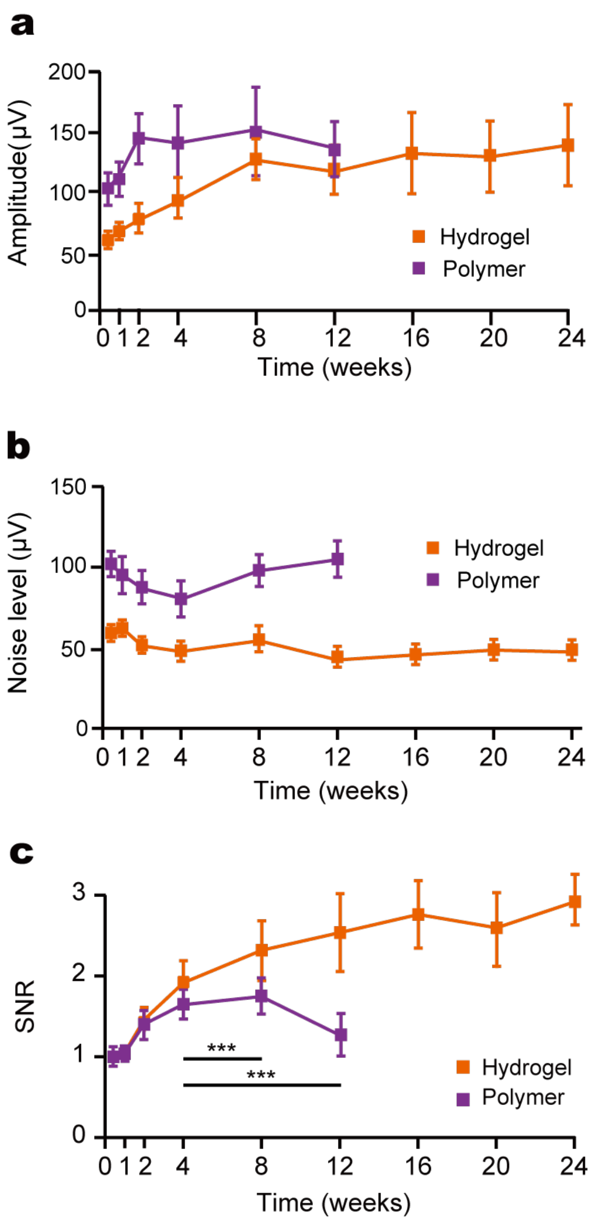


**Supplementary Fig. 21 | Endogenous electrophysiological signals recorded from various probes over time. a-c**, Amplitude of endogenous activities (a), background noise (b), and signal-to-noise ratio (SNR) (c) in the opto-electrophysiological experiments recorded from the hydrogel hybrid (orange) and polymer probes (purple) at various time points. Values in **a-c** represent the mean and the standard deviation (n = 8 for each, p = 0.001 and 0.0001 between 8 and 12 weeks, respectively, paired two-sided Student's t-tests).

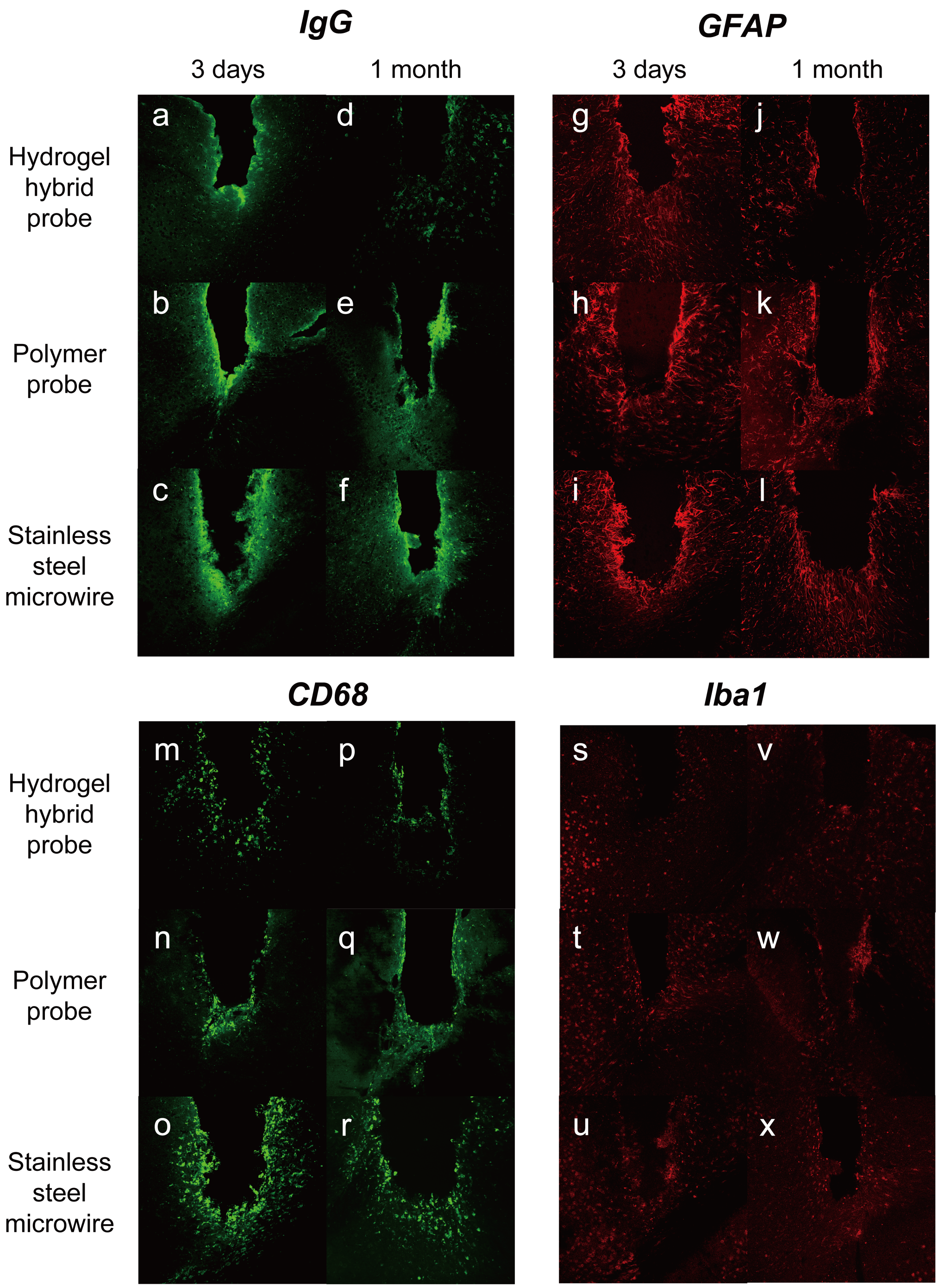


**Supplementary Fig. 22 | Immunohistochemistry images for probe implanted tissues. a-x**, Representative immunohistochemistry images tagged with IgG (a-f), GFAP (g-l), CD68 (m-r), and Iba1 (s-x) in the vicinity of the hydrogel hybrid probes, polymer probes, and stainless steel microwires for 3 days (a-c, g-i, m-o, s-u), and 1 month (d-f, j-l, p-r, v-x) following the implantation, respectively.

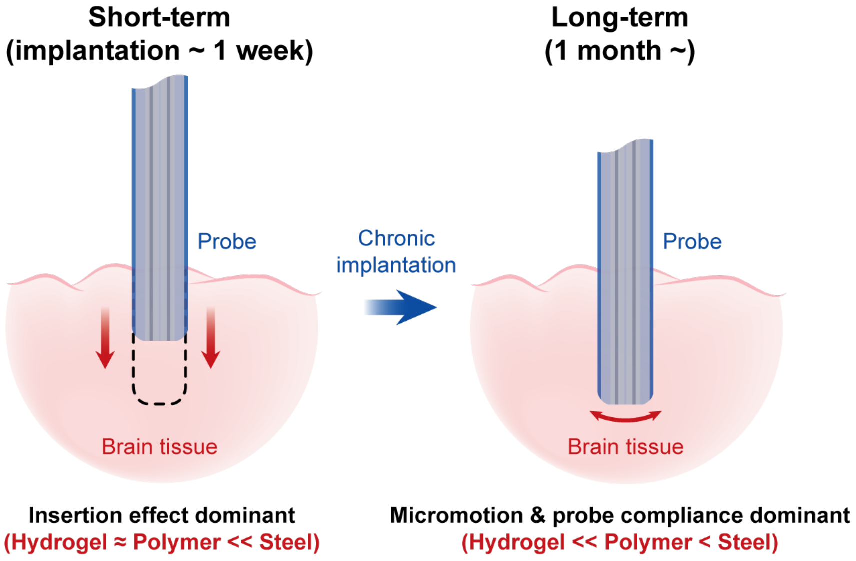


**Supplementary Fig. 23 | Device-tissue interactions in different time stages.** In the short-term after implantation, the insertion effect such as the acute tissue damage by probe insertion is mostly dominant. In the long-term after implantation, the chronic tissue response such as micromotion and subsequent probe-tissue interactions is more dominant.

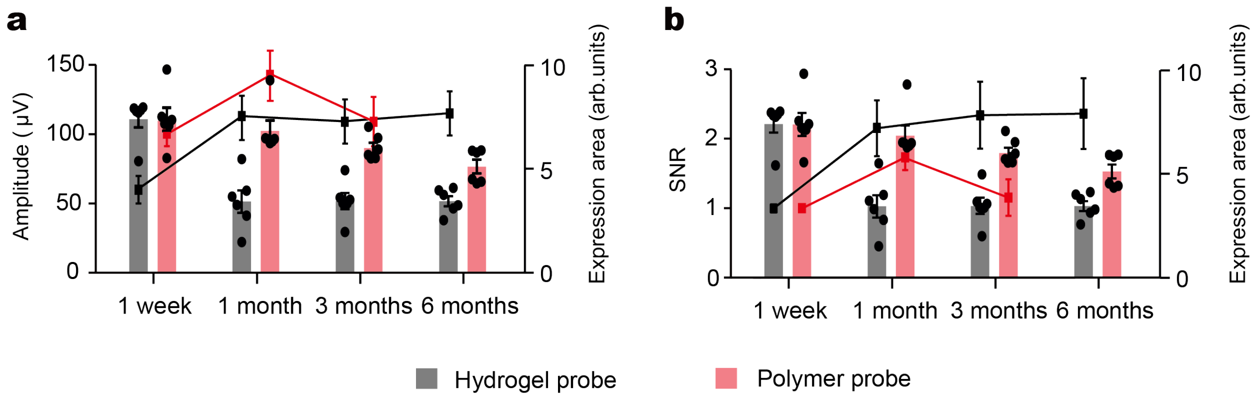


**Supplementary Fig. 24 | Relationship between foreign body response and quality of the recorded-signal. a**,**b**, Comparison between the average immunofluorescence area quantifying the presence of GFAP in the vicinity of the hydrogel hybrid (gray) and polymer probes (pink) and the amplitude (a) and the signal-to-noise ratio (SNR) (b) of the optically-evoked activities recorded with hydrogel hybrid (black) and the polymer probes (red) at various time points. Values in **a, b** represent the mean and the standard deviation (1 week and 1, 3, 6 months, $n$ = 6 for immuno-histochemical experiment and $n$ = 8 for opto-electrophysiological experiment).

**Supplementary Table 1 | Comparison of various candidate hydrogels for probe matrix.**

| Hydrogel | Shear modulus (kPa) | Fracture toughness (J $m^{-2}$) | Interfacial toughness (J $m^{-2}$) | Delamination failure in hybrid probe |
|---|---|---|---|---|
| PVA | 7.5 | 11 | 8 | Yes |
| Alginate | 4.3 | 24 | 5 | Yes |
| PEGDA-Alg | 8 | 120 | 21 | Yes |
| **PAAm-Alg** | **5.5** | **513** | **230** | **No** |

**Supplementary Table 2 | Bending stiffness of probes composed of different materials as calculated by FEA.** (Probe dimensions: 334 µm diameter and 3.4 mm length)

| Probe | Bending stiffness (N $m^{-1}$) |
|---|---|
| Stainless steel | 8191 |
| Silica | 2150 |
| Polycarbonate | 103 |
| Dried hybrid probe | 54 |
| Swollen hybrid probe | 7 |

**Supplementary Table 3 | The maximum number of units and the number of functional electrodes per probe.** The digits in the left parentheses represent the number of units recorded for each electrode. The digits in the right parentheses represent the number of working electrodes per probe.

| Max unit numbers (by pattern) | Functional channels considering overlapped pattern |
|---|---|
| 3<br>(2 / 1 / 0) | 2<br>(14) |
| 4<br>(2 / 1 / 1) | 3<br>(21) |
| 2<br>(1 / 1 / 0) | 2<br>(14) |
| 3<br>(0 / 1 / 2) | 2<br>(14) |
| 6<br>(2 / 2 / 2) | 3<br>(21) |
| 1<br>(0 / 1 / 0) | 1<br>(7) |
| 3<br>(1 / 1 / 0) | 3<br>(21) |
| 3<br>(0 / 1 / 1) | 2<br>(14) |

**Supplementary Table 4 | Mechanical properties for probe materials in FEA models.**

| Material | Young's modulus | Poisson's ratio |
|---|---|---|
| Stainless steel | 193 GPa | 0.265 |
| Silica | 50 GPa | 0.17 |
| Polycarbonate | 2.38 GPa | 0.37 |
| Tin | 50 GPa | 0.36 |
| Dried hydrogel | 50 MPa | 0.5 |
| Swollen hydrogel | 16.5 kPa | 0.5 |
| Brain | 9 kPa | 0.5 |

**Supplementary Table 5 | Antibodies and dilutions.**

| Primary Antibody | Secondary Antibody |
|---|---|
| Goat anti-GFAP (1:1000, Abcam ab53554 ) | Donkey anti-goat, Alexa Fluor 633 (1:1000, ThermoFisher A21082) |
| Goat anti-Iba1 (1:500, Abcam ab107159) | |
| Rabbit anti-CD68 (1:250, Abcam ab125212) | Donkey anti-rabbit, Alexa Fluor 488 (1:1000, ThermoFisher A21206) |
| Donkey anti-mouse IgG conjugated to Alexa Fluor 488 (1:1000, ThermoFisher A21202) | |

**Supplementary References**


1. Sun, J.-Y. et al. Highly stretchable and tough hydrogels. *Nature* **489**, 133 (2012).
2. VanDelinder, V. et al. Simple, benign, aqueous-based amination of polycarbonate surfaces. *ACS Appl. Mater. Interfaces* **7**, 5643-5649 (2015).
3. Coussot, G. et al. A rapid and reversible colorimetric assay for the characterization of aminated solid surfaces. *Anal. Bioanal. Chem.* **399**, 1061-1069 (2011).
4. Cha, C. et al. Tailoring hydrogel adhesion to polydimethylsiloxane substrates using polysaccharide glue. *Angew. Chem. Int. Ed.* **125**, 7087-7090 (2013).
5. Yuk, H., Zhang, T., Lin, S., Parada, G.A. & Zhao, X. Tough bonding of hydrogels to diverse non-porous surfaces. *Nat. Mater.* **15**, 190 (2016).
