# Supplementary material for: Adaptive and multifunctional hydrogel hybrid probes for long-term sensing and modulation of neural activity
Source: Nat Commun. 2021 Jun 8;12:3435. doi: 10.1038/s41467-021-23802-9 (PMC8187649; doi:10.1038/s41467-021-23802-9)
Supplement: Supplementary file 3 — Description of Additional Supplementary Files [file 41467_2021_23802_MOESM3_ESM.pdf]

Description of Additional Supplementary Files

Supplementary Movie 1 | Mises stress profiles in log scale for various probes during bending deformation.

Supplementary Movie 2 | Mises stress profiles in linear scale for various probes during bending deformation.

Supplementary Movie 3 | Insertion experiments of swollen and dried hybrid probes into the brain phantom.

Supplementary Movie 4 | Mises stress profiles within the brain tissue for various probes during micromotion.
