# Supplementary material for: Adaptive and multifunctional hydrogel hybrid probes for long-term sensing and modulation of neural activity
Source: Nat Commun. 2021 Jun 8;12:3435. doi: 10.1038/s41467-021-23802-9 (PMC8187649; doi:10.1038/s41467-021-23802-9)
Supplement: Supplementary file 8 — Reporting summary [file 41467_2021_23802_MOESM8_ESM.pdf]

nature research

Corresponding author(s): Polina Anikeeva and Xuanhe Zhao

Last updated by author(s): Mar 25, 2021

Reporting Summary

Nature Research wishes to improve the reproducibility of the work that we publish. This form provides structure for consistency and transparency in reporting. For further information on Nature Research policies, see Authors & Referees and the Editorial Policy Checklist .

Statistics

For all statistical analyses, confirm that the following items are present in the figure legend, table legend, main text, or Methods section.

n/a	Confirmed	
☐	☒	The exact sample size (*n*) for each experimental group/condition, given as a discrete number and unit of measurement
☐	☒	A statement on whether measurements were taken from distinct samples or whether the same sample was measured repeatedly
☐	☒	The statistical test(s) used AND whether they are one- or two-sided<br>*Only common tests should be described solely by name; describe more complex techniques in the Methods section.*
☐	☒	A description of all covariates tested
☐	☒	A description of any assumptions or corrections, such as tests of normality and adjustment for multiple comparisons
☐	☒	A full description of the statistical parameters including central tendency (e.g. means) or other basic estimates (e.g. regression coefficient) AND variation (e.g. standard deviation) or associated estimates of uncertainty (e.g. confidence intervals)
☐	☒	For null hypothesis testing, the test statistic (e.g. *F*, *t*, *r*) with confidence intervals, effect sizes, degrees of freedom and *P* value noted<br>*Give P values as exact values whenever suitable.*
☒	☐	For Bayesian analysis, information on the choice of priors and Markov chain Monte Carlo settings
☒	☐	For hierarchical and complex designs, identification of the appropriate level for tests and full reporting of outcomes
☒	☐	Estimates of effect sizes (e.g. Cohen's *d*, Pearson's *r*), indicating how they were calculated

Our web collection on statistics for biologists contains articles on many of the points above.

Software and code

Policy information about availability of computer code

Data collection	Tracking of mouse behaviour was performed using the EthoVision XT (Noldus).
Data analysis	GraphPad Prism 7 software was used to assess the statistical significance of all comparison studies; ABAQUS 2016 was used for finite element analysis; ImageJ (ver. 1.8.0_172) was used to quantify the area of fluorescence in immunohistochemistry images.

For manuscripts utilizing custom algorithms or software that are central to the research but not yet described in published literature, software must be made available to editors/reviewers. We strongly encourage code deposition in a community repository (e.g. GitHub). See the Nature Research guidelines for submitting code & software for further information.

Data

Policy information about availability of data

All manuscripts must include a data availability statement. This statement should provide the following information, where applicable:

- Accession codes, unique identifiers, or web links for publicly available datasets
- A list of figures that have associated raw data
- A description of any restrictions on data availability

All data supporting the findings of this study are available within the Article and its Supplementary Information. The raw data generated in this study are available from the corresponding author upon reasonable request.

Field-specific reporting

Please select the one below that is the best fit for your research. If you are not sure, read the appropriate sections before making your selection.

Life sciences study design

All studies must disclose on these points even when the disclosure is negative.

Sample size	Sample sizes were determined on the basis of the previous research conducted in the same brain circuits (Ref. 35 of manuscript)
Data exclusions	No data were excluded from the analysis.
Replication	Key experiments were reiterated with representatives from each group with similar observations across iterations.
Randomization	All the tests were performed with randomly allocated experimental groups.
Blinding	Experimenters were blind to subject treatment during data collection and analysis.

Reporting for specific materials, systems and methods

We require information from authors about some types of materials, experimental systems and methods used in many studies. Here, indicate whether each material, system or method listed is relevant to your study. If you are not sure if a list item applies to your research, read the appropriate section before selecting a response.

Materials & experimental systems

n/a	Involved in the study	
☐	☒	Antibodies
☒	☐	Eukaryotic cell lines
☒	☐	Palaeontology
☐	☒	Animals and other organisms
☒	☐	Human research participants
☒	☐	Clinical data

Methods

n/a	Involved in the study	
☒	☐	ChIP-seq
☒	☐	Flow cytometry
☒	☐	MRI-based neuroimaging

Antibodies

Antibodies used	goat anti-GFAP (ab53554, Abcam); goat anti-Iba1 (ab107159, Abcam); rabbit anti-CD68 (ab125212, Abcam); donkey anti-mouse IgG conjugated to Alexa Fluor 488 (A21202, ThermoFisher Scientific); donkey anti-goat labeled with Alexa Fluor 633 (A21082, ThermoFisher Scientific); donkey anti-rabbit labeled with Alexa Fluor 488 (A21206, ThermoFisher Scientific)
Validation	These are all commonly used antibodies. Statements regarding validation can be found at the manufactures website.

Animals and other organisms

Policy information about studies involving animals; ARRIVE guidelines recommended for reporting animal research

Laboratory animals	Male C57BL/6 mice aged 6 – 8 weeks (Jackson Laboratory) were used for the study. Mice were housed and maintained at 22 °C, 12 h light/dark cycle, and 50% humidity with provided the food and water ad libitum.
Wild animals	Study did not involve wild animals.
Field-collected samples	Study did not involve field-collected samples
Ethics oversight	All animal procedures were approved by the MIT Committee on Animal Care and carried out in accordance with the National Institutes of Health Guide for the Care and Use of Laboratory Animals.

Note that full information on the approval of the study protocol must also be provided in the manuscript.
